# Inhibition of Complement Will Substantially Reduce the Anemia of Malaria

**DOI:** 10.1155/jimr/3534692

**Published:** 2025-11-11

**Authors:** Ronald P. Taylor, Margaret A. Lindorfer

**Affiliations:** ^1^ Department of Biochemistry and Molecular Genetics, University of Virginia School of Medicine, Charlottesville, Virginia, USA, virginia.edu

## Abstract

There is compelling evidence that complement plays a major role in the elimination of nonparasitized erythrocytes (np‐Es) in the anemia of malaria. In fact, for every erythrocyte destroyed after infection by *Plasmodium falciparum* (Pf) or *Plasmodium vivax*, at least 10 np‐Es are cleared, most likely due to C3b opsonization. In malaria, complement is activated by three breakdown products released from lysed parasitized erythrocytes (p‐Es): hemin/hematin, the digestive vacuole (DV), and hemozoin. Both childhood anemia in malaria and post‐artesunate delayed‐hemolysis in individuals with malaria treated with artesunate can be ascribed to complement activation. These findings are important with respect to development of potential therapies for malarial anemia because there are now available FDA‐approved drugs that completely inhibit complement at the C3 activation step for several days at a time. We review key clinical findings and basic science investigations that implicate complement and in particular C3b opsonization as a significant factor in malarial anemia. Extension of these basic science studies to examine FDA‐approved drugs that block complement at the C3b opsonization step are needed to justify their potential utilization in treatment of malarial anemia. In particular, in vitro studies in blood cultures of Pf/*vivax* should determine whether deposition of C3 activation products on np‐Es can be completely prevented in the presence of complement inhibitors. If these experiments validate proof of concept, then extension of this paradigm to a clinical trial based on combined treatment with artesunate plus an FDA‐approved complement inhibitor would be indicated.

## 1. Introduction

Over the past 10 years the grim statistics for malaria have remained relatively constant; there are approximately 250 million cases of malaria each year, and a half million of these cases result in fatalities, mostly of African children [1–4]. Current treatments focus on artesunate and its derivatives which can rapidly kill the parasite [5–11]. These drugs are inexpensive and readily available, but it is clear that additional therapies are needed [5–11].

The life cycle of the malaria parasite in a parasitized erythrocyte (p‐E) is very well documented and results in the lysis of the infected E and the release of several by‐products that can activate complement [12, 13]. Also released are approximately 10–12 merozoites that can then infect additional E. Approximately 48 h later the newly infected p‐E are lysed in concert with the release of additional complement‐activating byproducts as well as additional merozoites. Although this reaction does indeed lead to loss of p‐E, there is now compelling evidence indicating that it is the clearance of non‐p‐E (np‐E) that is the principal cause of severe anemia in malaria due to *Plasmodium falciparum* (Pf) and *Plasmodium vivax* [14–19].

The mechanisms by which complement can be activated and controlled to provide focused defense against external threats have been described in detail [20, 21]. Both laboratory investigations and preclinical and clinical studies strongly support the hypothesis that complement activation plays a major role in the clearance of np‐E in malaria. The availability of FDA‐approved complement inhibitors offers an opportunity to test this hypothesis directly. On this basis we now propose in vitro experiments to provide a first and necessary step toward the development of a more efficacious treatment for malarial anemia.

## 2. Dichotomy: Levels of Parasitemia Versus Severity of Anemia

Numerous reports have indicated that the level of parasitemia in individuals with malaria is simply too low to account for the massive clearance of E that occurs. A particularly well‐focused study by Collins et al. [22] used archival data to retrospectively examine this phenomenon in 98 individuals who were infected with *Plasmodium vivax* in a treatment for syphilis. They reported that “the decreases in Hb” (i.e., E) “concentrations were far greater than can be accounted for by parasitic activity.” For example, in one representative individual, Hb levels decreased from 14 to 9 g/dL upon malaria infection but the maximum parasitemia was 10,000/μL. Based on a presumed E count of approximately 5 × 10^6^/μL, the decrease in Hb levels due to removal of p‐E would have been less than 0.03 g/dL.

Egan et al. [23] examined the dynamics of E destruction and levels of parasitemia in a nonhuman primate model in which the animals were first immunized against Pf. The immunized animals were then injected with p‐E from a donor infected monkey. As a result of the prior immunization, parasitemia was quite low in the monkeys receiving the injections. However, there was considerable loss of np‐E in several of the animals, strongly suggesting that the np‐E were being removed by a separate mechanism other than that due to direct infection by the parasites.

A comparable study in a murine model of malaria also revealed a remarkable quantitative dichotomy between the level of parasitemia (low) and the loss of E (high). Evans et al. [24] used biotinylated E as a surrogate to monitor np‐E in this mouse model. Semi‐immune mice were infected with *Plasmodium berghei* ANKA and when parasites could be demonstrated in the bloodstream (at very low levels) the mice were injected IV with EZ‐LinkSulfo‐NHS‐biotin, to rapidly label all the circulating E with biotin. Ten days later the investigators found that although the E levels were reduced by a factor of two, all of the biotinylated E (generated by the injection 10 days earlier) had been cleared from the circulation. This indicated that newly synthesized E were appearing in the bloodstream, but the E that were present 10 days earlier had all been cleared. As the levels of parasitemia were quite low throughout the experiment, there must be an alternative mechanism other than the lysis of p‐E to explain the complete loss of biotinylated E.

A similar “pulse‐chase” study was reported by Coleman et al. [25] who found that ^51^Cr‐labeled E had reduced lifetimes when they were infused into rats infected with very low levels of *Plasmodium berghei* (NK‐65 strain). The studies of Ekvall et al. [17] of patients with malaria also indicated that there was no evidence for substantial intravascular lysis of np‐E based on observing very low levels of free Hb in the bloodstream and urine.

## 3. Complement Activation: C3B Opsonization

One of the first demonstrations of the possible importance of complement in the anemia of malaria was reported in 1979 by Facer et al. [26–28]. They found that circulating E in children with malaria were Coombs positive and the most prevalent opsonin on the E was C3d. Their prescient measurements included demonstration of phagocytosis of np‐E by monocytes in the bloodstream. By 1979 it was well‐known that E in the circulation that are opsonized with C3 activation fragments are subject to phagocytosis by macrophages in the liver and spleen [29–31]. Facer et al. [26] suggested, based on their findings, that “it is likely these reactions contribute to the pathogenesis of the anemia in falciparum malaria”.

The reports by Facer et al. [26] did not rapidly promote a groundswell of investigations related to complement and malaria. However, in 2001 Goka et al. [32] found very similar patterns in children with malaria, also based on Coombs positivity. They stated: “We have shown a reproducible association between the degree of C3d binding to the E membrane and the severity of malaria in African children with falciparum anemia.”

Our laboratory demonstrated that reaction of E in NHS with hematin, an E breakdown product associated with malaria, leads to substantial covalent deposition of C3b fragments on the E, which are closely associated (colocalized) with E CR1 [33, 34]. Moreover, those E with the most complement receptor 1 (CR1) acquired the most C3b fragments, and if this reaction occurs in the bloodstream, then the E with the highest levels of CR1 would be the E most likely to be first cleared, thus, giving rise to the observations of Stoute, Waitumbi, and then Oyong et al. [35–40]. These investigators found that circulating E taken from children with malaria had low levels of CR1, and of decay accelerating factor (DAF), and C3 activation fragments could also be demonstrated on the E.

**Figure 1:**
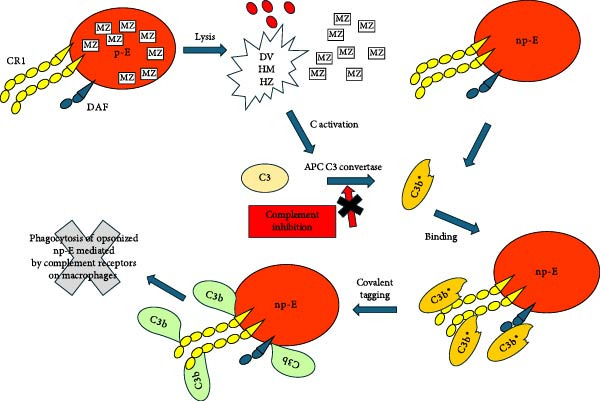
A parasitized erythrocyte lyses, and merozoites (MZ) are released, as well as E fragments and complement activating agents, including the digestive vacuole, (DV), hematin (HM), and hemozoin (HZ). Complement is then activated, principally by the APC, and nascent C3b (C3b ^∗^) is generated which can be chelated by CR1 or DAF. The nascent C3b then forms a covalent bond (C3b) with acceptor amino or hydroxyl groups on or very close to CR1 or DAF. C3b can be degraded to iC3b and C3d, and the opsonized E are then removed from the circulation by fixed tissue macrophages that recognize C3 activation fragments (C3b and iC3b). Inhibition of C3 activation by blocking the APC should prevent opsonization and subsequent phagocytosis of the np‐E.

Lysis of p‐E in the circulation in malaria will lead to massive local release of several complement‐activating agents (hemin/hematin, digestive vacuole [DV], and hemozoin) [41–43]. The nascent activated C3b ^∗^ that is generated should be captured by and covalently deposited on or quite close to CR1 and DAF on np‐E which are then subject to phagocytosis by fixed tissue macrophages (Figure 1). We postulate that a similar reaction occurs in mouse models of malaria, where the nascent C3b covalently deposits on or very close to the complement regulatory protein Crry/p65 expressed on mouse E [44].

The C3 fragments covalently bound to np‐E include C3b, iC3b, and C3d. Receptors for C3b (CR1) and iC3b (CR3) on phagocytes are well characterized and most likely play the major role in removing the opsonized E [29, 30, 45, 46]. Whether np‐E in which the C3 fragments have been degraded to C3d are also removed from the bloodstream in malaria is not clear. The work of Atkinson and Frank in complement opsonization models (not malaria) suggests that E that are opsonized with C3d persist in the circulation [29, 30]. Moreover, there is considerable evidence that after treatment of chronic lymphocytic leukemia patients with complement‐fixing CD20 mAbs, circulating B cells that have lost CD20 due to trogocytosis and are opsonized with only C3d persist in the circulation [47]. Therefore, we speculate that the np‐E in the circulation of malaria patients that are only opsonized with C3d could represent E in which the C3b/iC3b was degraded to C3d before the E could be cleared from the circulation.

## 4. The Entire Complement Pathway is Activated in Malaria

The 2015 report of Berg et al. [48] is particularly noteworthy because it included both in vitro experiments and analyses of blood samples taken from malaria patients. The in vitro results indicated that both hemin and hemozoin activated complement all the way to the terminal complex, generating soluble C5b‐9 (sC5b‐9). Moreover, sC5b‐9 was also detected in the bloodstream of malaria patients, and its levels were correlated with the malaria severity score. Although these measurements did not include quantitation of C5a, it also must be generated based on demonstration of sC5b‐9, which is produced upon scission of C5 to C5a and C5b [45, 49].

Complement activation by‐products can also synergize to increase clearance of C3b‐opsonized np‐E. It is well established that C5a, generated during complement activation, upregulates phagocytic receptors, such as CR3 (monitored based on CD11b) on granulocytes and monocytes [45, 49]. Thus, there can be a cooperative or synergistic effect: Products released from E lysed by the malaria parasite promote deposition of C3 activation fragments on np‐E, and receptors (CR3) on phagocytic cells for these ligands are upregulated, thus increasing the level of phagocytosis of the C3b‐opsonized np‐E. Long before these studies, Sheagren et al. [50] reported in 1970 that reticuloendothelial function is enhanced in human malaria. It is most likely that this very early observation can be attributed to the upregulation of CRs on phagocytic cells.

A group led by Bhakdi examined blood cultures of PF and found that DV released from lysed and infected E activated both the alternative pathway of complement (APC) as well as the clotting cascade, clearly implicating DV in malaria pathogenesis [41]. Their extension of these studies to a rat model reinforced the findings and also demonstrated, both in vitro and in vivo, that low molecular weight dextran sulfate could block both complement activation and coagulation, thus providing a possible path forward for treatment.

## 5. The Threshold for Activation of the APC

Seminal investigations of the mechanism of activation of the APC demonstrated a threshold effect due to its autocatalytic nature [51, 52]. That is, in the absence of initiators, due to reaction with water, C3 activation and C3b generation occurs continuously at low levels, and the reaction will be subject to additional control by the complement control proteins (CCPs) on cells. However, in the presence of complement activators, after sufficient C3b is covalently bound to a substrate to reach a threshold, then activation and deposition of C3b increases exponentially [51, 52]. It is reasonable to postulate that after artesunate treatment for malaria, a number of np‐E will have covalently acquired sufficient C3b to be slightly below the threshold. Then, in the course of normal circulation and low‐level complement activation over the next few days, some of these E will acquire additional C3b to reach the key threshold, and this will be followed by more C3b deposition and subsequent extravascular clearance of these C3 fragment‐opsonized np‐E.

## 6. Comparison of Malaria to Paroxysmal Nocturnal Hemoglobinuria (PNH)

Thorough analyses of the dynamics of E opsonization and clearance in patients with PNH indicates that E with normal levels of CCP are not tagged with C3 fragments; only the bona fide PNH E, which have low levels of CCP CD55 and CD59, are tagged. That is, during intravascular hemolysis in untreated PNH, the non‐PNH E (normal levels of CD55 and CD59) are not C3b‐opsonized as is the case for np‐E in malaria [53]. We suggest that this is because lysis of the PNH E does not occur in a brief, concerted, and orchestrated (explosive?) fashion every 48 h as is the case in malaria. In addition, hemozoin and the DV, two of the breakdown products from p‐E in malaria (that activate C), are not generated in PNH, thus further decreasing the possibility for “innocent bystander” C3b opsonization of normal E in PNH.

## 7. Delayed Hemolysis After Treatment With Artesunate

Artesunate and its derivatives have been proven to work quite effectively in the treatment of malaria, but there has been an increasing number of reports that a subfraction (~1/3) of treated children and adults who receive artesunate treatment experience anemia several days after the parasites are cleared [54–59]. The etiology of this delayed‐hemolysis syndrome is under investigation, but there is now evidence that the E of patients, who experienced this phenomenon were Coombs positive only for C3d, thereby indicating that complement is involved. Indeed, Camprubi et al. [54] suggested that this implication of complement was “more than a coincidence.”

## 8. Complement Inhibition as an Adjunct Therapy for Malarial Anemia

Our hypothesis is that the covalent deposition of C3 activation fragments on or close to CCP CR1 and DAF on E constitutes the most important mechanism for loss of np‐E in malaria. In vitro studies indicate that this reaction, mediated by hematin in the presence of NHS, can be completely blocked with several complement inhibitors, including mAb 3E7 (blocks the APC) and compstatin (blocks C3 activation) [52, 60]. Moreover, the reaction also does not occur in factor B‐depleted serum, in which activation of the APC is abrogated [33, 34, 52]. Additional evidence that np‐E can take up C3b fragments in malaria was reported by Waitumbi’s group [39]. They prepared cultures of Pf with NHS and E and then isolated the supernatants from these cultures. They found that in the presence of NHS, the supernatants from these cultures were able to promote deposition of C3 activation fragments on naïve E, providing additional evidence for C3b opsonization of np‐E mediated by products released from lysed p‐E.

On the basis of the clinical and basic science observations we have cited and the likely importance of complement in these phenomena, we suggest that targeted inhibition of complement at the C3 activation step (Figure 1) has real potential for the treatment of malarial anemia in combination with agents that kill the parasite, such as artesunate. We note that artesunate also inhibits complement activation, but it is cleared from the circulation much too rapidly (half life 1 h) [61] as it is currently used to have an adequate effect in blocking complement [62]. FDA‐approved complement inhibitors that could be examined in combination with artesunate include Pegcetacoplan [63], Iptacopan [64], and Danicopan [65]. Recently Pegcetacoplan was demonstrated to quantitatively block complement activation in the bloodstream of normal individuals for up to 4 days based on a single iv injection [66].

We suggest that there are in vitro experiments that would provide clear and convincing rationales for the use of the candidate complement inhibitors. Following the report of Korir et al. [39], E should be cultured for varying periods of time with either Pf or *Plasmodium vivax*, in the presence of NHS. The washed E can then be examined by flow cytometry for C3b deposition based on use of protocols which can distinguish p‐E from np‐E [67]. These baseline experiments should establish optimum conditions to mediate C3b deposition on the np‐E. Then, the experiments should be repeated in the presence and absence of the complement inhibitors cited above. Use of low molecular weight dextran sulfate [41] as well as heparin (inexpensive) as possible complement inhibitors should be included [68, 69]. Positive controls for inhibition of C3b deposition should include mAb 3E7, compstatin, and fB‐depleted serum [34]. If the candidate FDA‐approved inhibitors successfully block C3b deposition on np‐E, this would provide substantial motivation for moving on to pre‐clinical studies and ultimately to a clinical trial. A first choice for a clinical trial would likely focus on adults with malaria who receive artesunate therapy. The key endpoint would be reduction/elimination of post‐artesunate hemolytic anemia in individuals receiving the complement inhibitor.

## 9. Conclusions and Future Considerations

We have set forth a rational strategy to be followed to evaluate the role of complement in malarial anemia with the goal of making use of complement inhibitors for therapy. Estimates of yearly malaria deaths directly attributable to anemia are uncertain but are in the range of 25%–50% [70, 71] out of a total of approximately ½ million [1–4]. If this approach were to have even modest success, its impact could be substantial in terms of lives saved.

We recognize that there are many other questions that must be addressed with respect to the use of complement inhibitors in the treatment of malarial anemia. These uncertainties include economic factors and clinical issues that will be quite important if this approach is to be implemented for African children. However, our focus at this time must be on the basic science and tests for proof of concept. It is not unreasonable to expect that if complement inhibition is found to be effective in the treatment of any form of malarial anemia, then this could provide the motivation to address the economic challenges as well.

**Nomenclature**
APC: Alternative pathway of complement
C3b ^∗^: Nascent C3b with the thioester bond exposed
CCP: Complement control proteins
CR1: Complement receptor 1 (CD35)
DV: Digestive vacuole
DAF: Decay accelerating factor (CD55)
E: Erythrocytes
Hb: Hemoglobin
IV: Intravenous
HM: Hematin
HZ: Hemazoin
MZ: Merozoites
Pf: *Plasmodium falciparum*
np‐E: nonparasitized erythrocytes
p‐E: parasitized erythrocytes
PNH: Paroxysmal nocturnal hemoglobinuria.

## Conflicts of Interest

The authors declare no conflicts of interest..

## Funding

This article was written with no external funding.

## Data Availability

The data sharing is not applicable to this article as no new data were created or analyzed in this study.
